# Mining of immunological and prognostic-related biomarker for cervical cancer based on immune cell signatures

**DOI:** 10.3389/fimmu.2022.993118

**Published:** 2022-10-21

**Authors:** Nana Wang, Abiyasi Nanding, Xiaocan Jia, Yuping Wang, Chaojun Yang, Jingwen Fan, Ani Dong, Guowei Zheng, Jiaxin Ma, Xuezhong Shi, Yongli Yang

**Affiliations:** ^1^ Department of Epidemiology and Biostatistics, College of Public Health, Zhengzhou University, Zhengzhou, Henan, China; ^2^ Department of Pathology, Harbin Medical University Cancer Hospital, Harbin, Heilongjiang, China; ^3^ Department of Gynecology, Harbin Medical University Cancer Hospital, Harbin, Heilongjiang, China

**Keywords:** cervical cancer, immunotherapy, biomarkers, tumor immune microenvironment, immunohistochemistry

## Abstract

**Background:**

Immunotherapy has changed the therapeutic landscape of cervical cancer (CC), but has durable anti-tumor activity only in a subset of patients. This study aims to comprehensively analyze the tumor immune microenvironment (TIME) of CC and to mine biomarkers related to immunotherapy and prognosis.

**Methods:**

The Cancer Genome Atlas (TCGA) data was utilized to identify heterogeneous immune subtypes based on survival-related immune cell signatures (ICSs). ICSs prognostic model was constructed by Cox regression analyses, and immunohistochemistry was conducted to verify the gene with the largest weight coefficient in the model. Meanwhile, the tumor immune infiltration landscape was comprehensively characterized by ESTIMATE, CIBERSORT and MCPcounter algorithms. In addition, we also analyzed the differences in immunotherapy-related biomarkers between high and low-risk groups. IMvigor210 and two gynecologic tumor cohorts were used to validate the reliability and scalability of the Risk score.

**Results:**

A total of 291 TCGA-CC samples were divided into two ICSs clusters with significant differences in immune infiltration landscape and prognosis. ICSs prognostic model was constructed based on eight immune-related genes (IRGs), which showed higher overall survival (OS) rate in the low-risk group (*P*< 0.001). In the total population, time-dependent receiver operating characteristic (ROC) curves displayed area under the curve (AUC) of 0.870, 0.785 and 0.774 at 1-, 3- and 5-years. Immunohistochemical results showed that the expression of the oncogene (FKBP10) was negatively correlated with the degree of differentiation and positively correlated with tumor stage, while the expression of tumor suppressor genes (S1PR4) was the opposite. In addition, the low-risk group had more favorable immune activation phenotype and higher enrichment of immunotherapy-related biomarkers. The Imvigor210 and two gynecologic tumor cohorts validated a better survival advantage and immune efficacy in the low-risk group.

**Conclusion:**

This study comprehensively assessed the TIME of CC and constructed an ICSs prognostic model, which provides an effective tool for predicting patient’s prognosis and accurate immunotherapy.

## Introduction

Cervical cancer (CC) is the fourth most commonly diagnosed cancer and the leading cause of cancer deaths in women (1). Persistent infection with high-risk human papilloma virus (HPV) is a major causative factor in the progression of CC. With the continuous improvement of HPV vaccination rate, the growth trend of CC incidence has slowed down (2). However, due to distant metastasis and local recurrence after treatment, the prognosis of CC is not very ideal (3, 4).

Immunotherapy includes immune checkpoint blockade (ICB), immune cell therapies, cancer vaccines, and lysing viruses that target various types of immune cells and have dramatically changed the treatment landscape of many solid tumors (5). However, clinical trials have revealed that ICB could only exhibit durable antitumor activity in some patients with CC (6). Therefore, precise immunotherapy and accurate efficacy prediction of patients by immunotherapy-related biomarkers has manifested clinical research priorities.

Tumor immune microenvironment (TIME) in CC is characterized by high levels of immunogenicity and immune cells infiltration, suggesting that an in-depth study of TIME may be critical for tumor prognosis and treatment (7). Wang et al. (8) identified the PD-1 ^+^ DC density of the TIME might be a diagnostic factor in predicting the best beneficiaries of PD-1/PD-L1 blockade immunotherapy in CC. Furthermore, Walayat Shah et al. (9) found that reversal of the CD4/CD8 ratio of tumor-infiltrating lymphocytes and CD4 ^+^ FOXP3 ^+^ regulatory T cells high ratio were significantly associated with clinical prognosis in CC. Therefore, the use of computational methods to quantify TIME may provide more advanced prognostic biomarkers, which may reveal additional novel targets for chemotherapy and immunotherapy in CC patients.

This study explored the TIME of CC based on immune cell signatures (ICSs) and mined eight immune-related genes (IRGs). Biological function, immune cell infiltration, and immunotherapy-related biomarkers were mined to identify ideal immunotherapeutic subgroups for CC. The highlighted results provided methodological and technical support to achieve precision immunotherapy for CC.

## Materials and methods

### Data acquisition and processing

Fragments per kilobase million (FPKM) data of CC (N = 307) including gene expression profiles, somatic alteration data, and clinical data were downloaded from the Cancer Genome Atlas (TCGA) Genomic Data Commons (GDC) data portal (https://portal.gdc.cancer.gov/). The TCGA database is based on tissue specimens for high-throughput sequencing. The FPKM data were translated into transcripts per kilobase million (TPM). Duplicate recorded samples and overall survival (OS) time or survival status unavailable were excluded. Ultimately, 291 CC patients from the TCGA cohort were enrolled in the analyses, including 241 squamous cell carcinomas, 46 adenocarcinomas, and 4 adenosquamous carcinomas. Specific clinical information on CC patients was supplemented in **Data Files S1**.

These 184 ICSs were based on the aggregation of databases such as ImmPort, CIBERSORT, and ImSig (10). Detailed information was listed in **Data Files S2**. The normalized enrichment score (NES) generated by single sample gene set enrichment analysis (ssGSEA) with the R package “GSVA” (version 1.42.0) was considered as the infiltrate level of each ICS (11). In this study, only 183 ICSs were evaluated for follow-up analyses due to lack of some marker genes in the transcriptome atlas.

**Figure 1:**
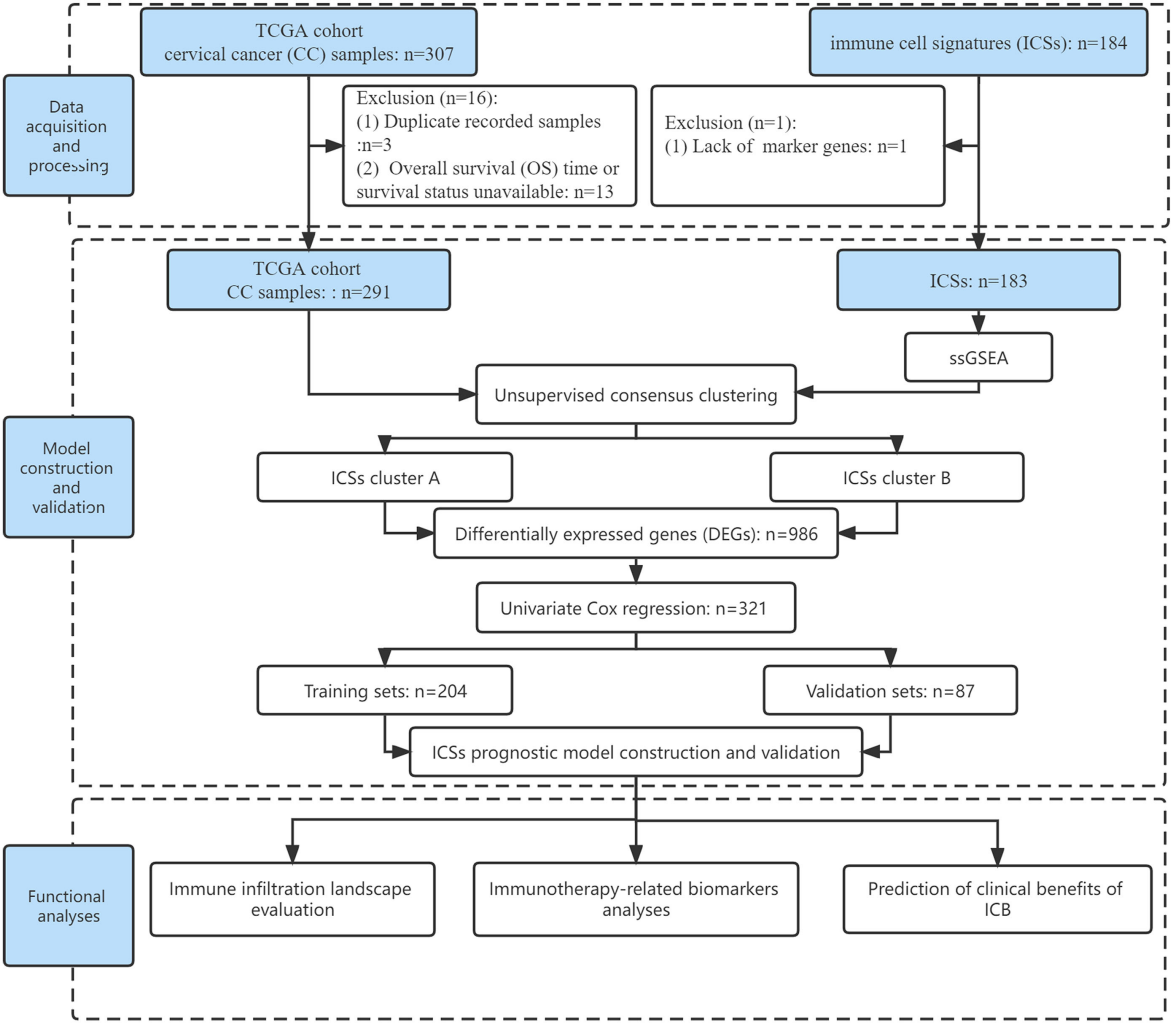
Flow chart of the study.

Meanwhile, the IMvigor210 cohort (http://research-pub.gene.com/IMvigor210CoreBiologies/) (N = 348) were also enrolled to validate our findings using the R package “IMvigor210CoreBiologies”, which are patients with metastatic urothelial cancer (mUC) receiving PD-L1 inhibitor (12). Raw count was also translated to TPM to represent gene expression in the IMvigor210 cohort. In addition, to expand our findings in gynecologic tumors, ovarian cancer (OC) and endometrial cancer (EC) data were downloaded and processed from the TCGA database. Finally, 374 OC patients and 539 EC patients were included. The flow of this study was shown in **Figure 1**.

### Unsupervised consensus clustering for survival-related ICSs

The univariate Cox regression was applied to obtain survival-related ICSs (*P*< 0.050). Subsequently, according to the infiltrate levels of survival-related ICSs, hierarchical agglomerative cluster of CC patients was performed using R package “ConsensusClusterPlus”. This algorithm was repeated 1,000 times to obtain stable classification. The differentially expressed genes (DEGs) between ICSs clusters were analyzed with false discovery rate (FDR)< 0.050 and absolute fold-change > 2, which was implemented by employing the R package “limma”. To identify the genomes and pathways enriched by DEGs, the Gene Ontology (GO) and Kyoto Encyclopedia of Genes and Genomes (KEGG) pathway functional enrichment analyses were performed using the R package “clusterProfiler” (version 4.2.2).

### ICSs prognostic model construction and validation

In the TCGA cohort, the univariate Cox regression was applied to obtain survival-related genes from DEGs (*P*< 0.050). Then, the TCGA-CC samples were randomly divided into training (n = 204) and validation (n = 87) sets at 7:3. Least absolute shrinkage and selection operator (LASSO) and multivariate Cox regression analyses were used to construct an ICSs prognostic model in the training set. The calculation formula of Risk score was as follows:


$$\text{Risk score}=\sum \beta_{i}\times ExpGene_{i}$$

In the formula, *β_i_* was defined as the coefficient of genes correlated with survival and ExpGene*_i_* was the expression value of the corresponding gene in each sample. The cut-off value was determined by the “surv_cutpoint” function of the R package “survminer”, which calculates statistics based on maximally selected rank statistics. The principle of this function to determine the optimal cutoff value is to obtain the two groups with the most statistically significant difference in survival rates through multiple simulations.

### Immunohistochemistry

Tumor tissues from a total of 23 CC patients were obtained from the Harbin Medical University Cancer Hospital. These samples were obtained from patients who underwent radical surgery, and all patients were untreated prior to radical resection. Specific clinical information is supplemented in **Data Files S3**. All specimens were collected in accordance with the ethical standards of the Committee for Human Experimentation. The expressions of S1PR4 and FKBP10 on the CC tissue were performed by immunohistochemistry (IHC) staining. Tissue sections were incubated with a primary antibody against S1PR4 or FKBP10 at 4°C overnight and then incubated with horseradish peroxidase combined with goat anti-rabbit antibody (PV-6001, ZSGB) at room temperature for 30 mins. Tissue sections were stained using DAB and counterstained with hematoxylin. The results of the experiment were analyzed by two doctors and two pathologists. The rules are as follows: Immunoreactive score (IRS) = SI (staining intensity) × PP (percentage of positive cells). SI was assigned as: 0 = negative; 1 = weak; 2 = moderate; 3 = strong. PP was defined as 0 = 0%; 1 = 0–24.9%; 2 = 25–49.9%; 3 = 50–74.9%; 4 = 75–100%. All of the included patients were dichotomized into two groups based on the median score.

### Immune infiltration landscape evaluation

To further explore the immune infiltration landscape in two prognostic subgroups, multiple immune-related algorithms were utilized, such as the ESTIMATE, CIBERSORT, and MCPcounter algorithms (13–15). In addition, we also analyzed the distribution of immune subtypes in high and low-risk groups, which were categorized in previous studies (16, 17). To further reveal the underlying mechanisms of CC, gene set variation analysis (GSVA) was performed using the R package “GSVA” (adjusted *P*< 0.050). The gene set “c2.cp.kegg.v7.4.symbols” was downloaded from Molecular Signatures Database (MSigDB, http://www.broad.mit.edu/gsea/msigdb/).

### Immunotherapy-related biomarkers analyses

The main biomarkers for the prediction of immune efficacy included immune mark genes, immune function characteristics, tumor mutational burden (TMB), histocompatibility complex (MHC) molecules, chemokines, cytolytic activity (CYT), and stimulator of interferon genes (STING) (18–20). Therefore, we further explored the differences in these immunotherapy-related biomarkers between high and low-risk groups.

### Immunotherapeutic response prediction

We downloaded the immunophenoscore (IPS) from the cancer immunome atlas (TCIA) (https://tcia.at/home) to predict responses to ICB (21). IPS was calculated based on the expression of MHC molecules, immunomodulators, effector cells (ECs) and suppressor cells (SCs). It included four types of scores, ips_ctla4_pos_pd1_pos, ips_ctla4_pos_pd1_neg, ips_ctla4_neg_pd1_pos, and ips_ctla4_neg_pd1_neg, to better predict the efficacy of anti-CTLA-4 and anti-PD-1 antibodies. IMvigor210 cohort and two gynecologic tumor (TCGA-OC and TCGA-EC) cohorts were also used to validate the predictive value of Risk score for immunotherapy.

### Statistical analyses

All statistical analyses were performed with R software (version 4.1.3). The Kaplan–Meier plotter was employed to depict survival curves. The Wilcoxon test was carried out to compare the difference between two groups, and the correlation coefficient was computed using the Spearman analyses. Two-tailed *P*< 0.050 was deemed statistical significance.

## Results

### Survival and immunological characterization between ICSs clusters

**Figure 2:**
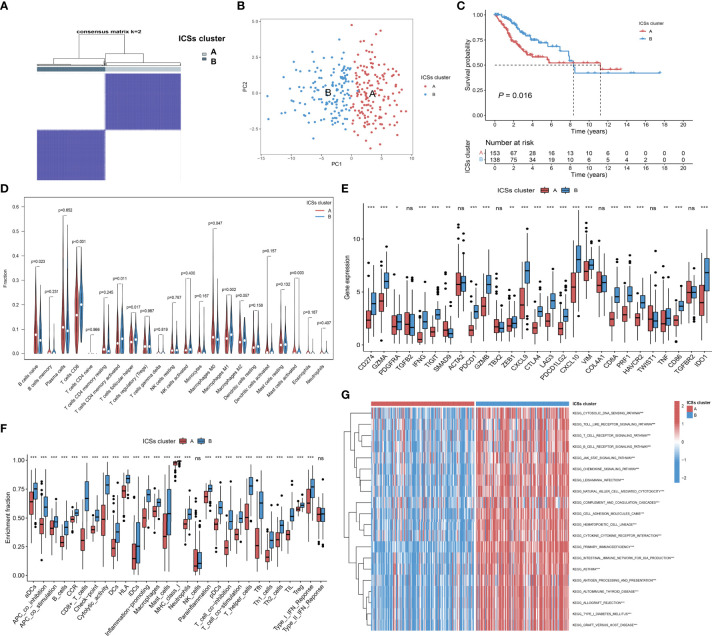
Determination of immune cell signatures (ICSs) subtypes. **(A)** Consensus matrix of the TCGA-CC cohort with appropriate k values (k = 2). **(B)** PCA validation of clustering results. **(C)** Kaplan-Meier curves of OS for CC patients in both ICSs clusters (*P* = 0.016). **(D)** Violin plots of 22 tumor-infiltrating immune cell types of two ICSs clusters by CIBERSORT algorithm. **(E)** Differential analysis of 27 immune marker genes in two ICSs clusters (ns, not significant; *P< 0.050; **P< 0.010; ***P< 0.001). **(F)** Differences between two ICSs clusters in the degree of enrichment of indicator signals for specific immune functions (ns, not significant; *P< 0.050; **P< 0.010; ***P< 0.001). **(G)** Biological processes of two ICSs clusters using GSVA analysis. Heatmap colors indicate ICSs infiltrate levels, with red indicating high infiltrate levels and blue indicating low infiltrate levels. (ns, not significant; *P< 0.050; **P< 0.010; ***P< 0.001).

The TCGA cohort was divided into two clusters based on survival-related ICSs by using hierarchical agglomerative cluster (**Figure 2A**; **Figures S1A, B**). The accuracy of the clustering was verified using principal component analysis (PCA) (**Figure 2B**). Furthermore, Kaplan-Meier survival curves showed significant survival difference between two ICSs clusters (*P* = 0.016; **Figure 2C**). Differential correlation patterns of survival-related ICSs between two clusters were visualized as heatmap (**Figure S1C**). Enriched biological processes of ICSs clusters were summarized by **Figures S1D and S1E**, with specific data in the **Data Files S4**.

To furtherly clarify the intrinsic substrates leading to different survival outcomes between two ICSs clusters, we performed a series of immune correlation analyses. CIBERSORT algorithm revealed that ICSs cluster A was characterized by high naive B cells, M0 macrophages, activated mast cells infiltration, which might be the cause of poor prognosis. By contrast, ICSs cluster B was marked by high CD8 T cells, memory activated CD4 T cells, follicular helper T cells, and M1 macrophages infiltration (**Figure 2D**). The heatmap of correlation coefficient was generated to visualize the cellular interaction of the tumor-infiltrating immune cell types (**Figure S1F**). MCPcounter and ESTIMATE algorithms similarly demonstrated a higher immune infiltration of ICSs cluster B (**Figure S1G**; **Figure S1H**). In the subsequent analysis, we compared the differential expression levels of 27 common immune marker genes in two clusters (22, 23). The results exhibited that most of the immune marker genes were comprehensively elevated in the ICSs cluster B (**Figure 2E**). Furthermore, ssGSEA of specific gene sets demonstrated that the ICSs cluster B was highly active in multiple immune function pathways (**Figure 2F**), consistent with the results of the above immune correlation analyses. GSVA identified that the B cell receptor, T cell receptor, and JAK/STAT pathways were significantly activated in the ICSs cluster B (**Figure 2G**). The results were supplemented in the **Data Files S5**.

### ICSs prognostic model construction and validation

**Figure 3:**
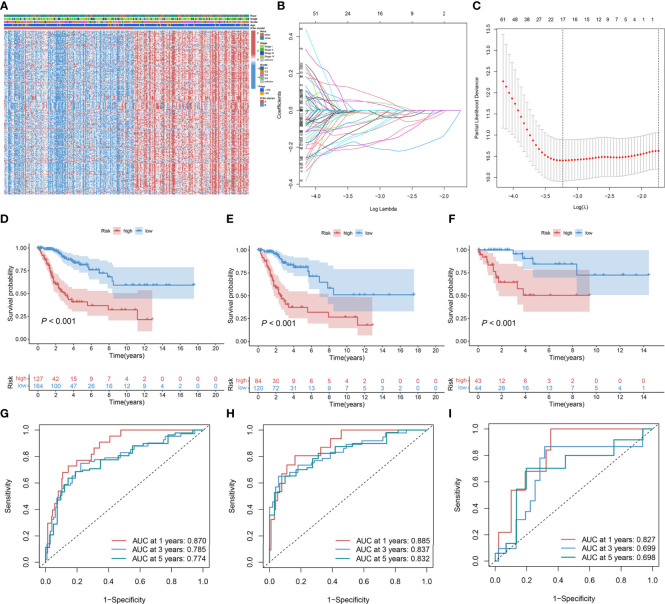
Construction and validation of ICSs prognostic model in the TCGA cohort. **(A)** Heatmap depicted the expression levels of survival-related DEGs in different ICSs clusters and the distribution of clinical traits of patients. The rows represent survival-related DEGs and the columns represent samples. **(B, C)** Determination of the number of survival-related DEGs into the multivariate Cox regression model by LASSO analyses. **(D–F)** Kaplan-Meier curves of OS for the high and low-risk groups in the total population, training set, and validation set (*P<* 0.001). **(G–I)** Time-dependent ROC curve in the total population, training set, and validation set.

The 986 DEGs (FDR< 0.050 and absolute fold-change > 2) were included in the univariate Cox regression model and 321 genes (*P*< 0.050) were found to be significant. Heatmap results displayed that the gene expression levels in ICSs cluster B were generally higher than that in ICSs cluster A (**Figure 3A**). All the 321 significant genes were incorporated into the LASSO and multivariate Cox regression model (**Figures 3B, C**). Finally, eight IRGs were contained in the ICSs prognostic model. The comprehensive Risk score was calculated as follows: Risk score = (0.13780 * CA9) + (0.29263 * FKBP10) + (-0.27821 * CKB) + (-0.40748 * GLIPR2) + (-0.42239 * ISG20) + (-0.42787 * S1PR4) + (-0.26784 * SDS) + (-0.20766 * VTCN1). Patients with CC were divided into high and low-risk groups using the cutoff value (1.27508) as the dividing line. The above results were showed in the **Data Files S6**.

In the total population, training and validation sets, the distribution curves and survival scatter plots indicate that patients with high-risk scores have a poorer prognosis (**Figures S2A–F**), and Kaplan-Meier survival curves demonstrated that patients showed a significant difference between high and low-risk groups in survival rate (*P*< 0.001; **Figures 3D–F**). In the total population, time-dependent receiver operating characteristic (ROC) curves showed that the ICSs prognostic model had a strong prognostic accuracy with the area under the curve (AUC) of 0.870 in 1 year, 0.785 in 3 years and 0.774 in 5 years (**Figure 3G**). The results of time-dependent ROC curves for the training and validation sets were shown in **Figures 3H, I**.

**Figure 4:**
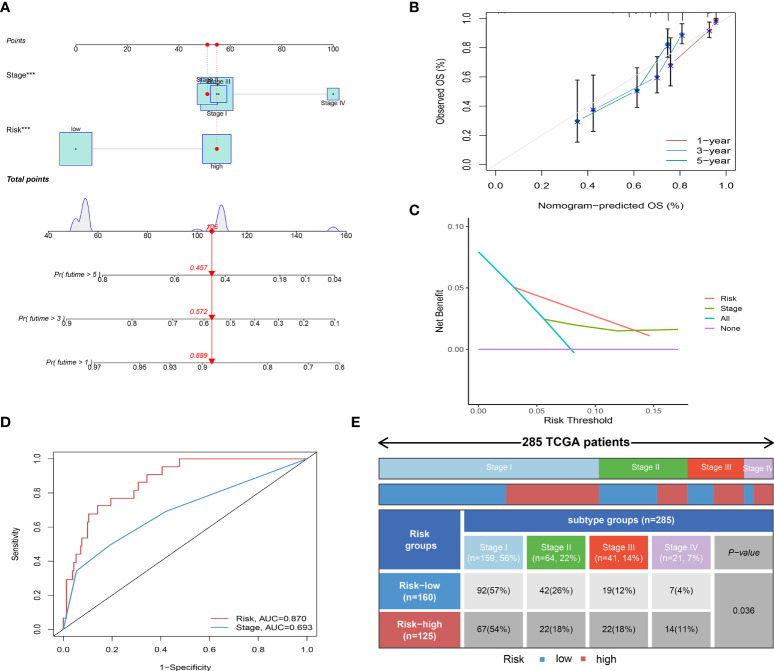
Independent prognostic analysis of Risk score. **(A)** Nomogram for predicting the probability of patient mortality at 1-, 3-, or 5- year OS based on two independent prognosis factors (***P< 0.001). **(B)** Calibration curves of the nomogram for predicting the probability of OS at 1-, 3-, or 5- year. **(C)** Decision curve analyses (DCAs) of the nomograms for 1-, 3-, or 5- year risk. **(D)** Time-dependent ROC curve of two independent prognosis factors. **(E)** Heatmap and table showing the distribution of Stage I-IV between high and low-risk groups (*P* = 0.036).

Univariate and multivariate Cox regression showed that Risk score and Stage were independent prognostic factors for CC (**Data Files S7**). By combining the independent prognostic factors, we constructed a nomogram that serves as a clinically relevant quantitative method by which clinicians could predict the mortality of CC patients (**Figure 4A**). In addition, calibration plots indicated that the performance of the nomogram was similar to that of the ideal model (**Figure 4B**). The decision curve analysis (DCA) also revealed that the nomogram had high potential clinical utility (**Figure 4C**). We also compared the AUC of the Risk score and Stage, and the results showed that the Risk score had better predictive power (**Figure 4D**). Finally, we compared the distribution of Stage between high and low-risk groups, as shown in **Figure 4E**, with a higher proportion of Stage I-II patients in the low-risk group.

### Immunohistochemical results of FKBP10 and S1PR4

**Figure 5:**
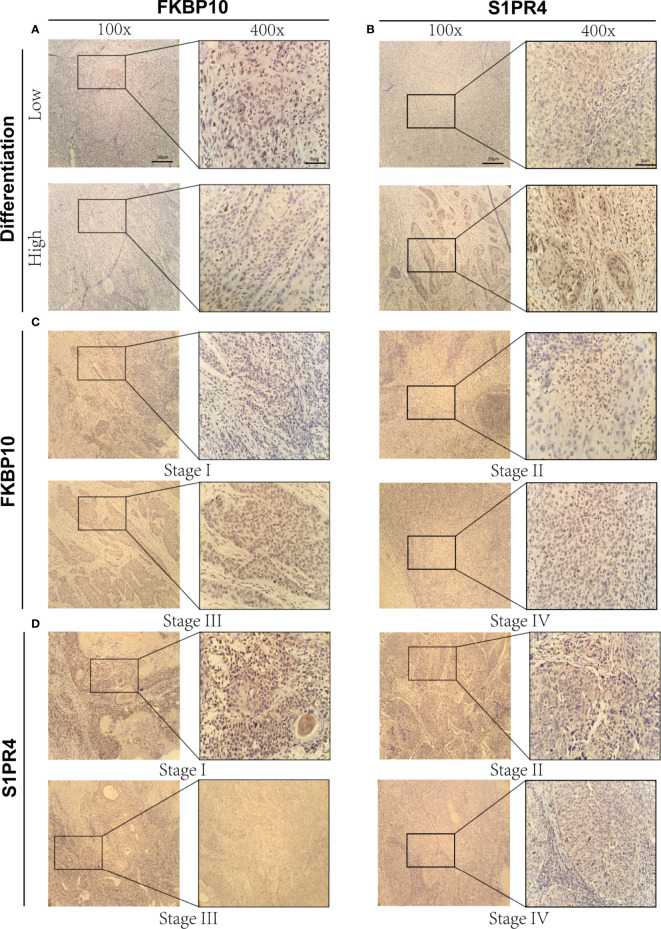
Immunohistochemical results of FKBP10 and S1PR4. **(A)** Immunohistochemical images of FKBP10 in high and low differentiation groups. **(B)** Immunohistochemical images of S1PR4 in high and low differentiation groups. **(C)** Immunohistochemical images of FKBP10 in four different tumor stages (Stage I-IV). **(D)** Immunohistochemical images of S1PR4 in four different tumor stages (Stage I-IV).

To verify our previous research results, IHC staining was performed on tissue samples collected from patients with CC to explore the expression of oncogenes (FKBP10) and tumor suppressor genes (S1PR4) with the largest weight coefficient in the ICSs prognostic model. **Figures 5A and B** show immunohistochemical images of FKBP10 and S1PR4 in different differentiation states. The immunohistochemical results showed that the expression level of oncogene FKBP10 was negatively correlated with the degree of differentiation, while the expression of tumor suppressor gene S1PR4 was the opposite. In addition, this study used immunohistochemical images of tissues at different stages to demonstrate that the expression level of FKBP10 gradually increased with increasing tumor stage, and that of S1PR4 gradually decreased with increasing tumor stage (**Figures 5C, D**). This evidence confirmed the expression of two key genes in CC tissues, and our results had a higher degree of confidence.

### Immune infiltration landscape in high and low-risk groups

**Figure 6:**
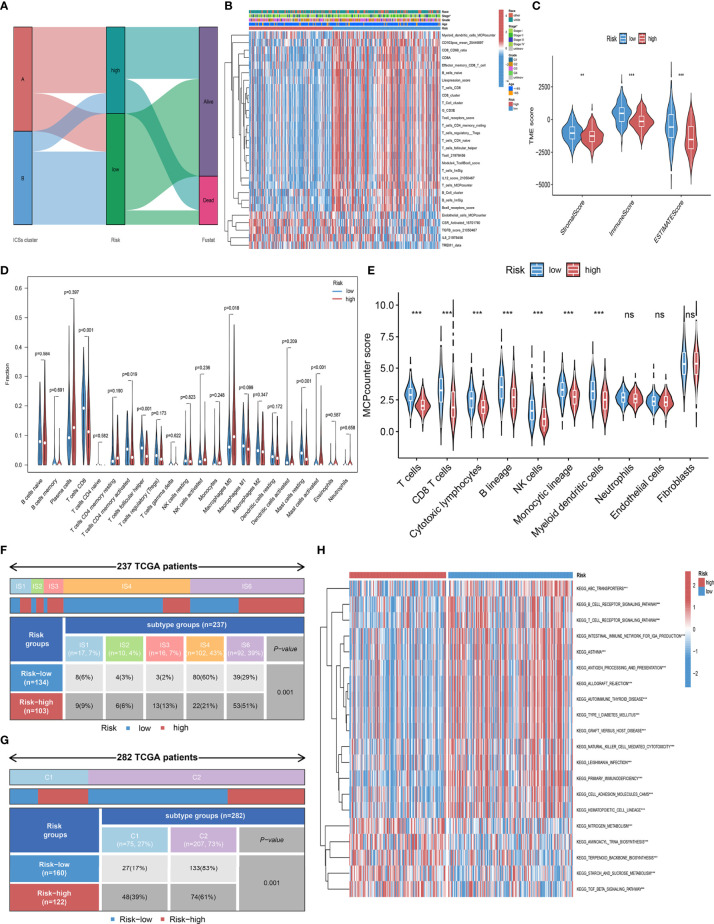
Immune infiltration landscape in the high and low-risk groups. **(A)** Alluvial diagram of the distribution with patients in different ICSs clusters, risk groups, and survival outcomes. **(B)** Heatmap depicted the infiltration of survival-related ICSs in the high and low-risk groups and the distribution of clinical traits of patients. The rows represent survival-related ICSs and the columns represent samples. **(C)** Differential analysis of immune score, stromal score and estimate score in the high and low-risk groups (ns, not significant; *P< 0.050; **P< 0.010; ***P< 0.001). **(D)** Violin plots of 22 tumor-infiltrating immune cell types of high and low-risk groups by CIBERSORT algorithm. **(E)** Violin plots of 10 tumor-infiltrating immune cell types of high and low-risk groups by MCPcounter algorithm (ns, not significant; *P< 0.050; **P< 0.010; ***P< 0.001). **(F)** Heatmap and table showing the distribution of pan- SCC immune subtypes (IS1, IS2, IS3, IS4, IS5, and IS6) between high and low-risk groups (*P* = 0.001). **(G)** Heatmap and table showing the distribution of immune subtypes (C1, C2, C3, C4, C5, and C6) between high and low-risk groups (*P* = 0.001). **(H)** Biological processes of high and low-risk groups using GSVA analysis. Heatmap colors indicate ICSs infiltrate levels, with red indicating high infiltrate levels and blue indicating low infiltrate levels. (ns, not significant; *P< 0.050; **P< 0.010; ***P< 0.001).

To further explore the correlation between the prognosis and TIME, we analyzed the immune infiltration landscape of CC. Alluvial diagram illustrated that most patients in ICSs cluster B have low Risk score and more alive status (**Figure 6A**; **Figure S3A**). In addition, we assessed the differential correlation pattern of prognosis-related ICSs in the high and low-risk groups, and the result was shown in **Figure 6B**. Further analyses showed that low-risk group had higher immune score, stromal score and estimate score (**Figure 6C**). CIBERSORT and MCPcounter algorithm displayed that the immune cell infiltrating types of the low-risk group were similar to ICSs cluster B, and the high-risk group was consistent with ICSs cluster A (**Figures 6D, E**). The association heatmap visualized a negative correlation between Risk score and multiple tumor-infiltrating immune cells (**Figure S3B**). The distribution of immune subtypes showed that the low-risk group was mainly distributed in IS4 and C2 types, which reflected that the low-risk group had a more favorable anti-tumor immune response (**Figures 6F, G**). GSVA was used to further explore the potential role of Risk score in biological processes, which identified that B cell receptor, T cell receptor and ATP-binding cassette (ABC) transporters pathways were significantly activated in the low-risk group (**Figure 6H**). Detailed results of GSVA were listed in the **Data Files S8**.

### Immunotherapy-related biomarkers differences between high and low-risk groups

**Figure 7:**
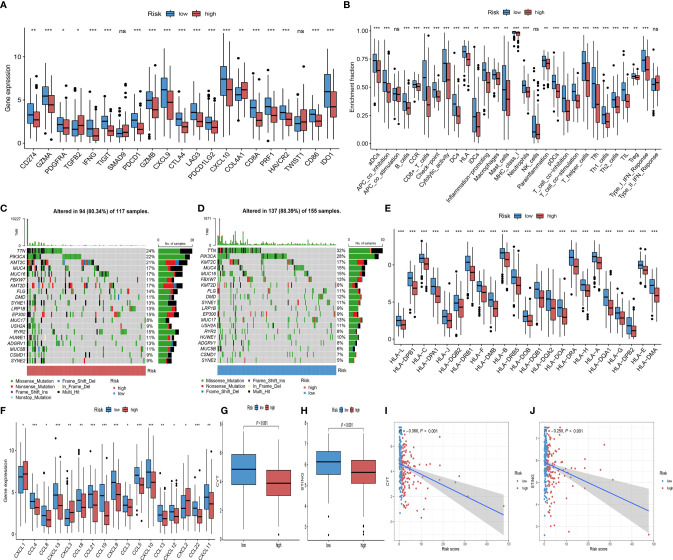
Comparison of immunotherapy predictive biomarker. **(A)** Differential analysis of 27 immune marker genes in the high and low-risk groups (ns, not significant; *P< 0.050; **P< 0.010; ***P< 0.001). **(B)** Differences between high and low-risk groups in the degree of enrichment of indicator signals for specific immune functions (ns, not significant; *P< 0.050; **P< 0.010; ***P< 0.001). **(C, D)** The waterfall diagram of the top 20 driver genes between the high **(C)** and low risk-score **(D)** of CC patients. **(E, F)** Differential analysis of MHC molecules **(E)** and chemokines **(F)** in the high and low-risk groups (ns, not significant; *P< 0.050; **P< 0.010; ***P< 0.001). **(G)** CYT difference in the high and low-risk groups (ns, not significant; *P< 0.050; **P< 0.010; ***P< 0.001). **(H)** STING difference in the high and low-risk groups (ns, not significant; *P< 0.050; **P< 0.010; ***P< 0.001). **(I)** Scatterplots depicting the correlation between Risk score and CYT (*R* = -0.380, *P*<0.001). **(J)** Scatterplots depicting the correlation between Risk score and STING (*R* = -0.250, *P*<0.001).

To further elucidate the effects of Risk score in the context of immunotherapy, we explored the associations between Risk score and several well-known immune marker genes. It was shown that immune marker genes were expressed at higher levels in the low-risk group (**Figure 7A**). We analyzed 29 immune function-related characteristics and found that the low-risk group had a more favorable immune activation phenotype, suggesting that they may have a more intense immune response (**Figure 7B**). Considering the great clinical significance of TMB for immunotherapy, we sought to explore the intrinsic correlation between TMB and Risk score. The “maftools” R package was carried out for assessing the distribution of somatic mutation in the high and low-risk groups, and depicted the top 20 driver genes with the highest alternative frequencies in **Figures 7C, D**. The heatmap of correlation coefficient demonstrated the interrelationship of the top 20 mutated genes in CC patients (**Figure S3C**). We found no difference in the prognosis of patients in the high and low TMB group (**Figure S3D**), as well as no significant correlation between TMB and Risk score (**Figures S3E**, **F**). In addition, we found that the expression levels of MHC molecules, and chemokines, which were responsible for the movement of immune cells, were comprehensively elevated in the low-risk group (**Figures 7E, F**). CYT and STING were relatively higher in the low-risk group (*P*< 0.001; **Figures 7G, H**), and Risk score was significantly negatively associated with relatively higher (**Figures 7I, J**). Taken together, these results suggested that Risk score based on eight IRGs may be potential predictors of CC immunotherapy efficacy.

### Prediction of clinical benefits of ICB

**Figure 8:**
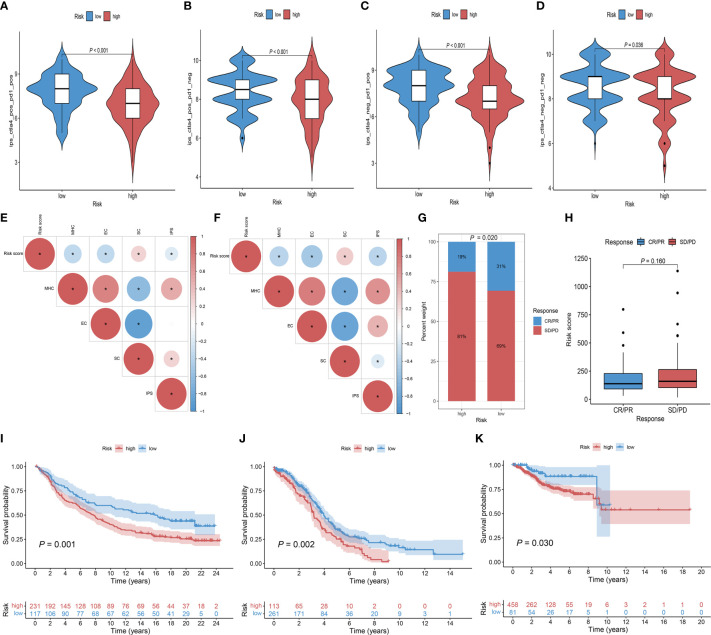
The role of Risk score in immunotherapeutic response prediction. **(A–D)** The distribution plot of ips_ctla4_pos_pd1_pos **(A)**, ips_ctla4_pos_pd1_neg **(B)**, ips_ctla4_neg_pd1_pos **(C)**, and ips_ctla4_neg_pd1_neg **(D)** scores in TCGA-CC cohort. **(E, F)** Intrinsic connection of Risk score and MHC, EC, SC, IPS score in TCGA-CC and IMvigor210 cohorts, with red indicating positive correlations and blue indicating negative correlations. The asterisks represented the statistical *P* value (*P< 0.050). **(G)** Distribution of clinical response rates for anti-PD-L1 immunotherapy in the high and low-Risk score groups in the IMvigor210 cohort (*P* = 0.020). **(H)** Risk score in groups with different anti-PD-L1 clinical response status (*P* = 0.160). **(I)** Kaplan-Meier curves for OS in the IMvigor210 cohort for the high and low-risk groups (*P* = 0.001). **(J)** Kaplan-Meier curves for OS in the TCGA-OC cohort for the high and low-risk groups (*P* = 0.002). **(K)** Kaplan-Meier curves for OS in the TCGA-EC cohort for the high and low-risk groups (*P* = 0.030).

To assess the ability of the Risk score as a biomarker for predicting clinical response to ICB treatment, we assessed the immunogenicity of two prognostic subgroups by IPS analyses. The low-risk group had higher ips_ctla4_pos_pd1_pos, ips_ctla4_pos_pd1_neg, ips_ctla4_neg_pd1_pos, and ips_ctla4_neg_pd1_neg scores in the TCGA-CC cohort (**Figures 8A–D**). In the TCGA-CC and IMvigor210 cohort, Risk score was negatively correlated with MHC scores, EC scores and IPS scores. Regarding the SC score, opposite results were obtained (**Figures 8E, F**). In the subsequent analyses, IMvigor210 cohort were assigned high and low-risk score. As shown in **Figures 8G, H**, while there was no statistical difference in Risk scores between patients with complete response (CR)/partial response (PR) and those with stable disease (SD)/progressive disease (PD), clinical response rates with anti-PD-L1 therapy were higher in patients with low-risk group than those with high-risk group (*P* = 0.020). Noteworthy, patients with low-risk score had a significantly better prognosis than those with high-risk score in the IMvigor210 and two gynecologic tumors (**Figures 8I–K**). In addition, IPS analyses indicated consistent results for the TCGA-EC cohort with the TCGA-CC cohort (**Figures S4A–D**), while only the ips_ctla4_pos_pd1_neg score was higher in the low-risk group of the TCGA-OC cohort (**Figures S4E–H**). These results indicated that patients in the low-risk group may have a better response to immunotherapy.

## Discussion

Immunotherapy, as a novel treatment strategy for CC, only benefit a minority of patients (24). In this study, we constructed ICSs prognostic model based on eight IRGs. Patients in the low-risk group had higher survival rates and immune activated cell infiltration. In addition, we revealed a greater enrichment of immunotherapy-related biomarkers in the low-risk group. Notably, we also evaluated the prognostic and immunotherapeutic role of the Risk score in the IMvigor210 and two gynecologic tumors cohorts.

Published work suggested that the TIME plays an important role in improving prognosis and mediating the therapeutic response to chemotherapy and immunotherapy in patients with CC (7). In this study, we categorized the patients with CC into two ICSs clusters. Our analyses indicated that ICSs cluster B with higher densities of CD4 ^+^ T cells, CD8 ^+^ T cells, and M1 macrophages, as well as higher immune score, were associated with patient prognosis, which is in line with the previous studies (25, 26). In addition, cluster B was enriched for more immunoreactive and signaling pathways compared to ICSs cluster A. Therefore, patients with ICSs cluster B may generate a more intense immune response. However, despite the higher degree of immune infiltration, the existing clinical studies reported lower response rates to immunotherapy in patients with CC, suggesting that there may be underlying molecular mechanisms in the anti-tumor process (27, 28).

In this study, we focused on the molecular characteristics that regulate the immune system in CC. We screened eight IRGs (CA9, FKBP10, CKB, GLIPR2, ISG20, S1PR4, SDS, VTCN1). Previous studies observed that genes such as CA9, CKB and GLIPR2 were strongly associated with the prognosis of cancer (29–31). FKBP10 and S1PR4 had the largest weight coefficients compared with other genes in the model, suggesting that the expression levels of these two genes have a greater impact on patient prognosis. FKBP10, an endoplasmic reticulum chaperone, coordinates protein translation to sustain lung cancer growth, but the mechanism of action in CC has not been elucidated (32). Bioinformatics analysis has shown that hypermethylation and low expression of S1PR4 are associated with poor prognosis of CC, but experimental verification is still lacking (33). In the present study, based on immunohistochemical experiments, we verified the differences in the expression levels of FKBP10 and S1PR4 in different differentiation states and different tumor stages, further demonstrating the plausibility of the results of this study. In conclusion, the ICSs prognostic model constructed based on eight IRGs quantified the risk of individual patients and effectively identified high-risk patients.

To further understand the immunological nature of the two prognostic subgroups, we analyzed their immune infiltration landscape. We found that the immune infiltration phenotype of the low-risk group was consistent with ICSs cluster B, suggesting that the low-risk group has a more favorable immune activation phenotype and is a “hot” tumor (34). In addition, we found more IS4 and C2 in the low-risk group of CC patients. IS4 had the highest T-cell and IFNγ gene expression as well as low-reactive stroma and TGFβ, and C2 had the highest M1/M2 macrophage polarization and strong CD8 signaling, which means a more favorable antitumor immune response in the low-risk group. In contrast, the high-risk group pooled more IS6 and C1, implying low inflammatory signaling and higher angiogenic gene expression (16, 17). These findings reveal an active immune response and lower tumor aggressiveness in the low-risk group and a suppressed immune response and greater tumor aggressiveness in the high-risk group.

Considering the individual heterogeneity of immunotherapy efficacy, there is an urgent need to investigate new therapeutic markers to identify ideal subgroups for CC immunotherapy. In the present study, we found that common biomarkers representing better immune efficacy were more enriched in the low-risk group, which is in line with our previous study (35, 36). However, the difference in TMB was not statistically significant in the high and low-risk groups, probably due to the lower level of TMB with the median index value was 1.908 (1.184-3.414). Previous studies have indicated that there is no significant difference in TMB between PD-L1-positive and PD-L1-negative subsets at lower TMB levels in CC cohorts (mean and median index value were 7.74 and 5.00, respectively), and therefore it may not be appropriate to investigate its application as a potential biomarker for immune checkpoint therapy at low overall TMB levels (37). To further validate the value of ICSs prognostic model, we evaluated the IPS scores in the high and low-risk groups, the results also demonstrated that patients in the low-risk group benefited more from immunotherapy (38). Analyses of the IMvigor210 cohort receiving anti-PD-L1 therapy also showed a better survival advantage and higher objective remission rates in the low-risk group as well. Gynecologic malignancies mainly include three major types of cervical cancer, ovarian cancer and endometrial cancer. It has been suggested that there may share a common molecular mechanism among the three gynecologic malignancies (39–41). Our findings for the other two gynecologic tumors were consistent, which may provide some references for further revealing the common molecular mechanisms of the three gynecologic tumors.

Although the present study suggests that the ICSs prognostic model may have clinical translational promise, there are still limitations. First, the relationship between prognostic models and immune efficacy was only preliminarily analyzed in the IMvigor210 cohort, which needs to be validated in other immunotherapy cohorts. Secondly, we only conducted IHC experiments for FPBK10 and S1PR4, and deeper mechanistic studies and clinical translation should be elucidated more systematically *in vitro* and *in vivo*.

In summary, we carried out a comprehensive assessment of the immune infiltration landscape in CC patients and screened the biomarkers based on immunotherapy relevance. In future studies, systematic evaluation of ICSs in tumor patients is important to achieve precision immunotherapy.

## Data availability statement

The original contributions presented in the study are included in the article/**Supplementary Material**. Further inquiries can be directed to the corresponding authors.

## Ethics statement

The studies involving human participants were reviewed and approved by Life Science Ethics Review Committee of Zhengzhou University. The patients/participants provided their written informed consent to participate in this study. Written informed consent was obtained from the individual(s) for the publication of any potentially identifiable images or data included in this article.

## Author contributions

Study design, NW and YY. Data collection, YW and JF. Data analyses, NW and CY. Collection, processing and analysis of specimens, AN and JM. Manuscript writing, NW. Manuscript review and revise, XJ and XS. All authors contributed to the article and approved the submitted version.

## Funding

This research was supported by the Key Scientific Research Project of Colleges and Universities in Henan Province (No. 23A330003; No 23B330001).

## Acknowledgments

All authors would like to thank the TCGA, IMvigor210 cohort and the specimen donors of this study, who provided data support for this study.

## Conflict of interest

The authors declare that the research was conducted in the absence of any commercial or financial relationships that could be construed as a potential conflict of interest.

## Publisher’s note

All claims expressed in this article are solely those of the authors and do not necessarily represent those of their affiliated organizations, or those of the publisher, the editors and the reviewers. Any product that may be evaluated in this article, or claim that may be made by its manufacturer, is not guaranteed or endorsed by the publisher.

## Supplementary material

Supplementary Data Sheet 1 Specific clinical information on TCGA-CC patients.

Supplementary Data Sheet 2 Names and marker genes for 184 immune cell signatures.

Supplementary Data Sheet 3 Clinical information of 23 CC patients from Harbin Medical University Cancer Hospital.

Supplementary Data Sheet 4 GO and KEGG results of differentially expressed genes.

Supplementary Data Sheet 5 Results of GSVA for two IGSs clusters.

Supplementary Data Sheet 6 Results of prognostic model construction, including univariate Cox regression, LASSO and multivariate Cox regression.

Supplementary Data Sheet 7 Independent prognostic validation in combination with clinical factors.

Supplementary Data Sheet 8 Results of GSVA for high and low-risk groups.

Supplementary Figure 1
**(A)** Cumulative distribution function (CDF) of consensus clustering for k = 2 to 9. **(B)** Relative change in area under the CDF curve, k = 2 to 9. **(C)** Heatmap depicted the infiltration of survival-related ICSs in different ICSs clusters and the distribution of clinical traits of patients. The rows represent survival-related ICSs and the columns represent samples. **(D)** GO enrichment analysis of DEGs in two ICSs clusters. **(E)** KEGG analysis of DEGs in two ICSs clusters. **(F)** Cellular interactions of the 22 tumor-infiltrating immune cell types. **(G)** Violin plots of 10 tumor-infiltrating immune cell types of two ICSs clusters by MCPcounter algorithm (ns, not significant; **P*< 0.050; ***P*< 0.010; ****P*< 0.001). **(H)** Differential analysis of immune score, stromal score and estimate score in two ICSs clusters (ns, not significant; **P*< 0.050; ***P*< 0.010; ****P*< 0.001).

Supplementary Figure 2
**(A–F)** The distribution of patients, Risk scores as well as survival status in the total population, training set, and validation set.

Supplementary Figure 3
**(A)** Risk score difference in different ICSs clusters. **(B)** Intrinsic connection of Risk score and 10 tumor-infiltrating immune cell types. The asterisks represented the statistical *P* value (**P*< 0.050). **(C)** Intrinsic connection of the top 20 driver genes in patients with CC. **(D)** Kaplan-Meier curves for the high and low TMB of patients. **(E)** TMB difference in the high and low-risk groups. **(F)** Scatterplots depicting the correlation between Risk score and TMB.

Supplementary Figure 4
**(A–F)** The distribution of patients, Risk scores as well as survival status in the total population, training set, and validation set.
