# Supplementary figures and images for: Mining of immunological and prognostic-related biomarker for cervical cancer based on immune cell signatures
Source: Front Immunol. 2022 Oct 21;13:993118. doi: 10.3389/fimmu.2022.993118 (PMC9634000; doi:10.3389/fimmu.2022.993118)

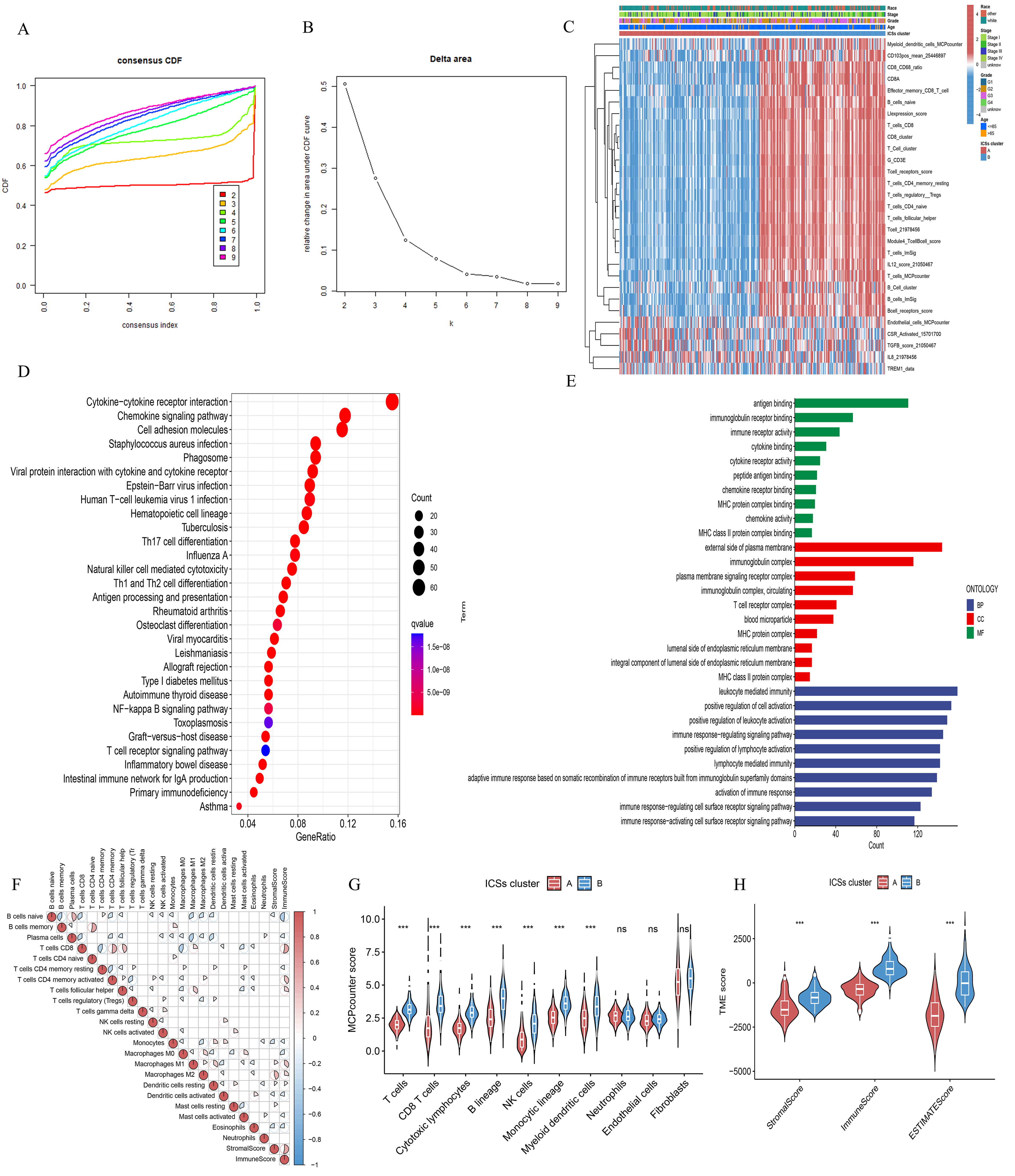

Supplement: Supplementary Figure 1 — (A) Cumulative distribution function (CDF) of consensus clustering for k = 2 to 9. (B) Relative change in area under the CDF curve, k = 2 to 9. (C) Heatmap depicted the infiltration of survival-related ICSs in different ICSs clusters and the distribution of clinical traits of patients. The rows represent survival-related ICSs and the columns represent samples. (D) GO enrichment analysis of DEGs in two ICSs clusters. (E) KEGG analysis of DEGs in two ICSs clusters. (F) Cellular interactions of the 22 tumor-infiltrating immune cell types. (G) Violin plots of 10 tumor-infiltrating immune cell types of two ICSs clusters by MCPcounter algorithm (ns, not significant; *P< 0.050; **P< 0.010; ***P< 0.001). (H) Differential analysis of immune score, stromal score and estimate score in two ICSs clusters (ns, not significant; *P< 0.050; **P< 0.010; ***P< 0.001). [file Image_1.tif]

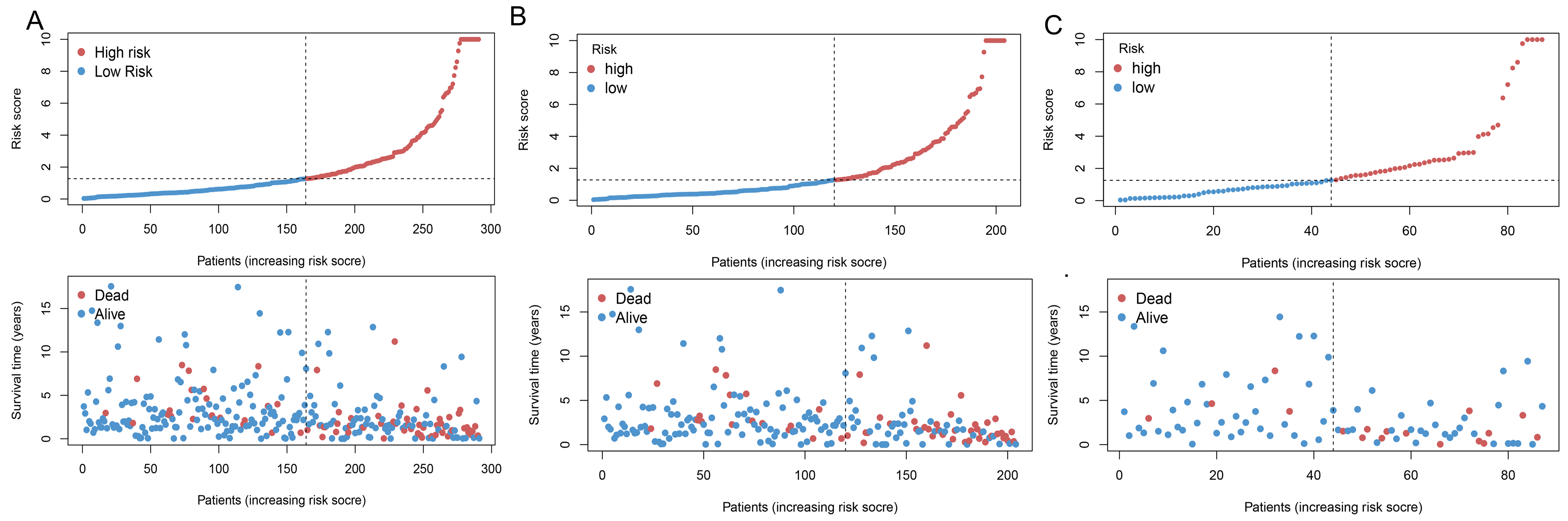

Supplement: Supplementary Figure 2 — (A–F) The distribution of patients, Risk scores as well as survival status in the total population, training set, and validation set. [file Image_2.tif]

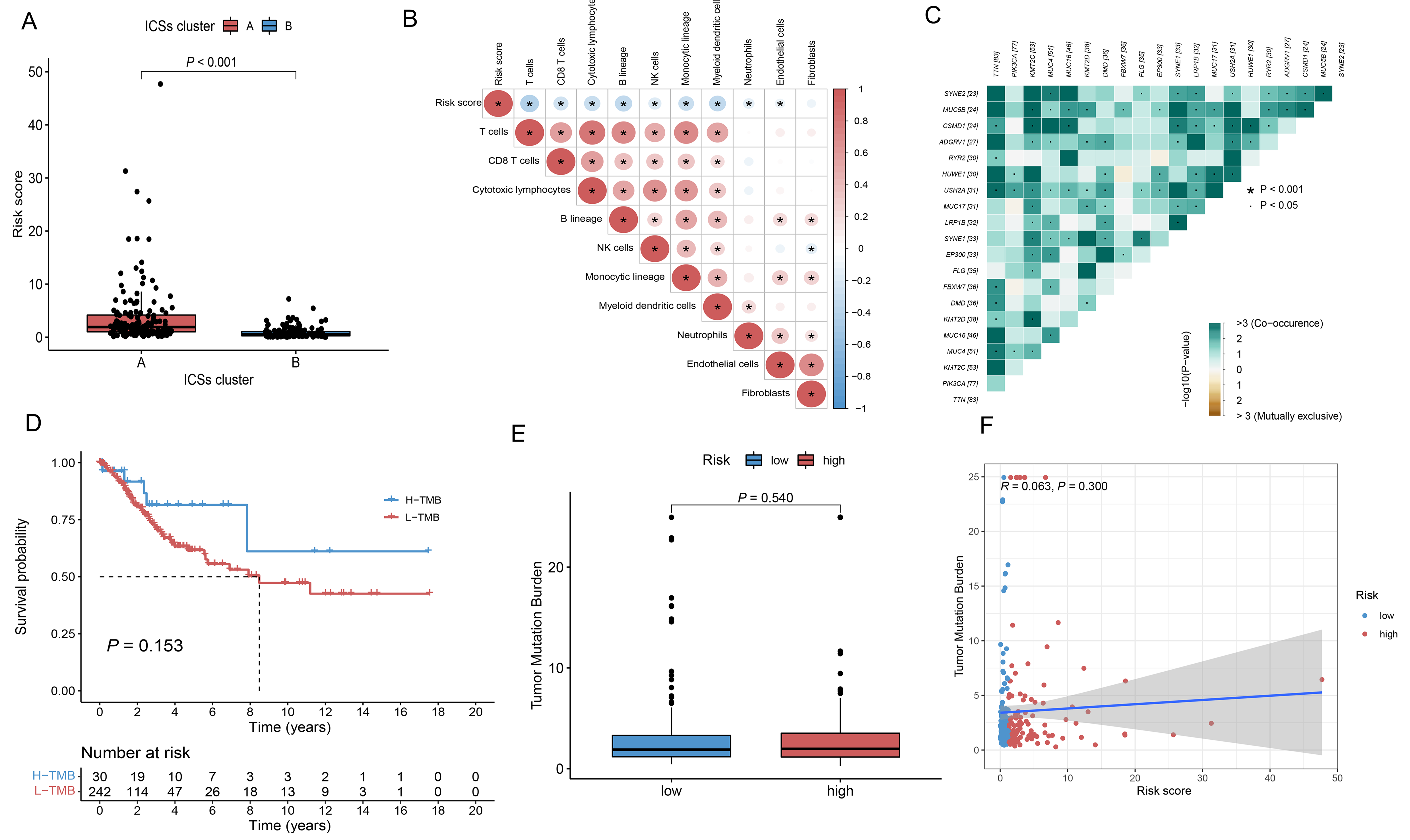

Supplement: Supplementary Figure 3 — (A) Risk score difference in different ICSs clusters. (B) Intrinsic connection of Risk score and 10 tumor-infiltrating immune cell types. The asterisks represented the statistical P value (*P< 0.050). (C) Intrinsic connection of the top 20 driver genes in patients with CC. (D) Kaplan-Meier curves for the high and low TMB of patients. (E) TMB difference in the high and low-risk groups. (F) Scatterplots depicting the correlation between Risk score and TMB. [file Image_3.tif]

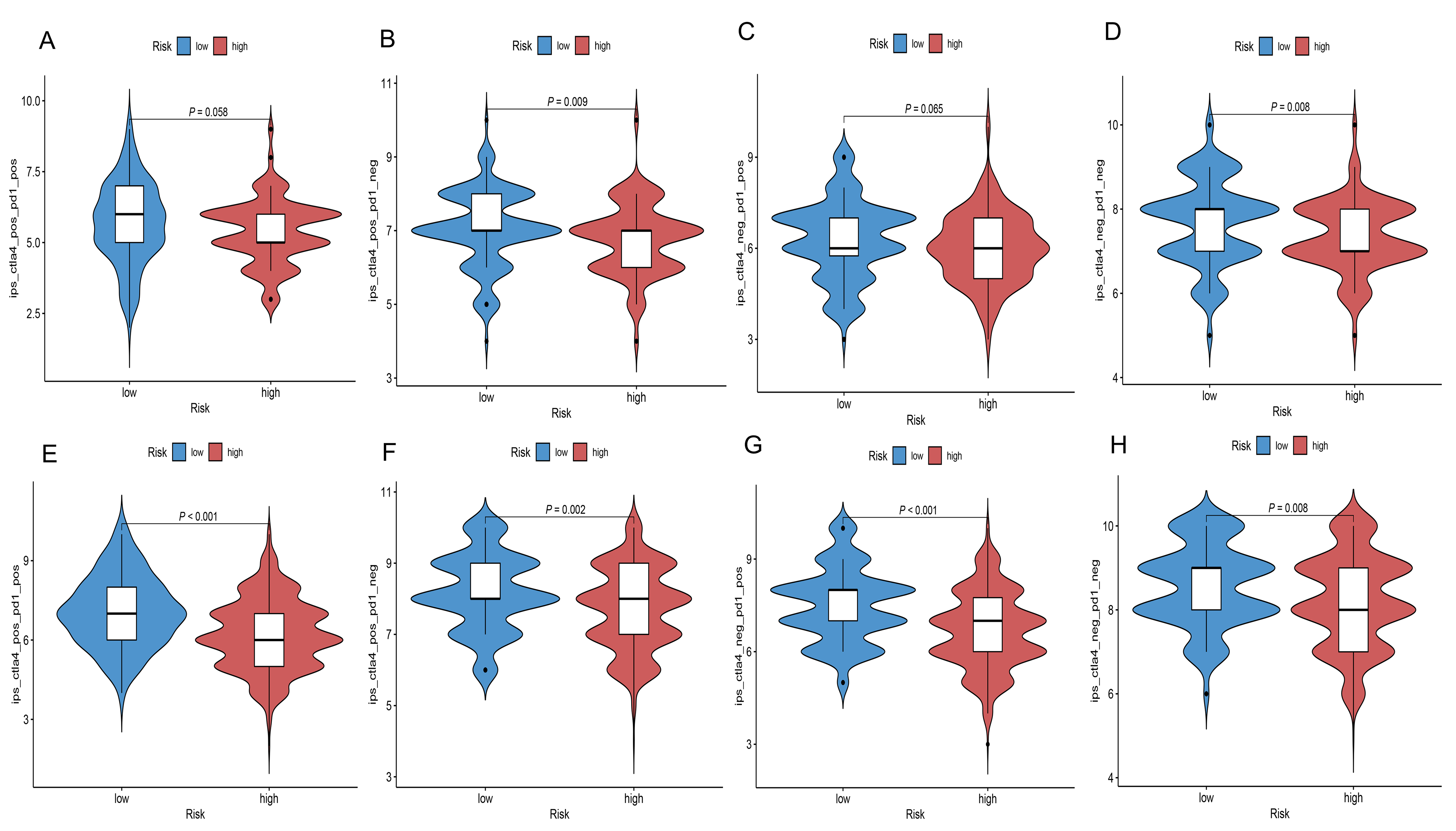

Supplement: Supplementary Figure 4 — (A–F) The distribution of patients, Risk scores as well as survival status in the total population, training set, and validation set. [file Image_4.tif]
